# Supplementary material for: Molecular decoupling of lineage identity and morphology in aggressive variant prostate cancer
Source: medRxiv. 2026 Jan 9:2026.01.07.26343520. Preprint. [Version 1] doi: 10.64898/2026.01.07.26343520 (PMC12803401; doi:10.64898/2026.01.07.26343520)
Supplement: Supplement 1 [file media-1.pdf]

Supplementary Figure 1

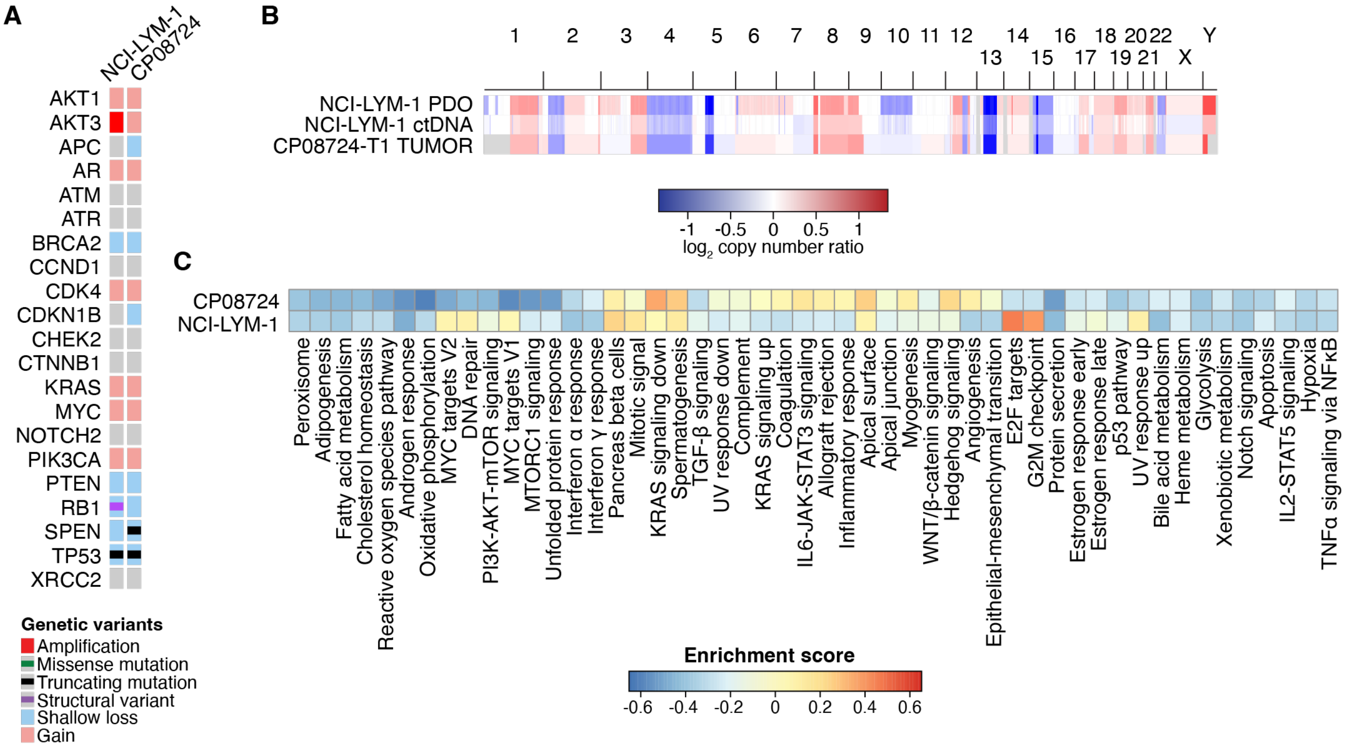

Supplementary Figure 1. Comparison of molecular features between NCI-LYM-1 and donor tumor biopsy. (A) Oncoprint depicting copy number and mutational status of selected prostate cancer genes NCI-LYM-1 and donor tumor biopsy. All mutations shown were curated for known oncogenic status. **(B)** Whole-genome somatic copy-number estimates derived from whole-genome sequencing NCI-LYM-1 and whole-exome sequencing of donor tumor biopsy. **C)** Heatmap depicting unsupervised nonparametric gene set variation analysis for NCI-LYM-1 and donor tumor biopsy transcriptomes projected against the mSigDB Hallmarks gene sets.
